# Supplementary material for: A graph neural network-based spatial multi-omics data integration method for deciphering spatial domains
Source: PLoS Comput Biol. 2025 Sep 30;21(9):e1013546. doi: 10.1371/journal.pcbi.1013546 (PMC12503342; doi:10.1371/journal.pcbi.1013546)
Supplement: S1 Text — (DOC) [file pcbi.1013546.s001.doc]

Supplementary information for ‘A graph neural network-based spatial multi-omics data integration model for deciphering spatial domains’

Congqiang Gao^1^, Chenghui Yang^2^ and Lihua Zhang^2,3*^

1 School of Cyber Science and Engineering, Wuhan University

2 School of Computer Science, Wuhan University

3 School of Artificial Intelligence, Wuhan University, Wuhan, 430072, China.

*Corresponding author(s).

E-mail(s): zhanglh@whu.edu.cn;

Contributing authors: [gaocongqiang@whu.edu.cn](mailto:gaocongqiang@whu.edu.cn); [2022282110116@whu.edu.cn](mailto:%202022282110116@whu.edu.cn)

**Supplementary Notes**

1. **Evaluation metrics**

We used five quantitative metrics to evaluate data integration performance of SpaMI and other comparative methods, including four supervised metrics, ARI, AMI, NMI, and homogeneity, and an unsupervised metric, Moran's I score.

**1.1 Adjusted Rand Index (ARI)**

ARI is a metric used to assess the consistency between clustering results and true labels. It is an improved version of the Rand Index (RI), which makes the results more reliable and comparable by adjusting for the effects of random factors. The Rand index is a measure of agreement between two clustering results. It is calculated by the formula:

$$RI=\frac{a+b}{a+b+c+d}$$

where $a$ is the number of pairs of samples belonging to the same class in both predictive clustering and true labeling; $b$ is the number of pairs of samples belonging to different classes in both predictive clustering and true labeling; $c$ is the number of pairs of samples belonging to the same class in predictive clustering, but belonging to different classes in true labeling; $d$ is the number of pairs of samples belonging to different classes in predictive clustering, but belonging to the same class in true labeling. However, the value of the RI may be high even if the clustering results are randomly assigned. In order to eliminate the effect of the random factor, the ARI was proposed. The formula for the ARI is:

$$ARI=\frac{RI-E\left[ RI \right]}{max(RI)-E\left[ RI \right]}$$

where, $E\left[ RI \right]$ is the expected value of the Rand index, $max(RI)$ is the maximum value of the Rand index.

**1.2 Adjusted Mutual Information (AMI) and Normalized Mutual Information (NMI)**

AMI and NMI are two metrics used to assess the consistency between clustering results and true labels. They are both based on the concept of Mutual Information (MI), but normalize or adjust the MI in different ways to eliminate the influence of random factors.

Mutual information is a measure of similarity between two clustering results. It reflects the amount of information shared between two clustering results. Mutual information is calculated by the formula:

$$MI\left( U, V \right)=\sum_{i=1}^{\left| U \right|} \sum_{j=1}^{\left| V \right|} P\left( i,j \right)\log\frac{P\left( i,j \right)}{P\left( i \right)P\left( j \right)}$$

where, $U$ and $V$ are true labeling and predictive clustering, respectively; $P(i,j)$ is the sample belonging to both true labeling categories $i$ and the predicted clustering category $j$; $P(i)$ and $P(j)$ are the probability that the sample belongs to the true labeling category $i$ and the predicted clustering category $j$, respectively. The range of values for the mutual information is $[0,+\infty)$, with higher values indicating higher agreement between two clustering results. In order to eliminate the effect of the number of categories in the clustering results, NMI has been proposed. NMI is achieved by normalizing the mutual information to the $[0,1]$ to make the results more comparable. NMI is calculated by the formula:

$$NMI=\frac{MI\left( U,V \right)}{\sqrt{H\left( U \right)H\left( V \right)}}$$

where $H(U)$ and $H(V)$ are the entropy of the true label $U$ and the predicted clusters $V$, respectively, calculated as:

$$H\left( U \right)=-\sum_{i=1}^{\left| U \right|} P\left( i \right)\log P\left( i \right)$$

$$H\left( V \right)=-\sum_{j=1}^{\left| V \right|} P\left( j \right)\log P\left( j \right)$$

The range of values of NMI is $[0,1]$, with higher values indicating higher consistency between the clustering results and the true labels.

Although NMI eliminates the effect of the number of categories, it can still be affected by random factors. To further eliminate randomness, AMI was proposed. AMI makes the results more reliable by adjusting the expected value of mutual information. AMI is calculated by the formula:

$$AMI\left( U,V \right)=\frac{MI\left( U,V \right)-E\left[ MI\left( U,V \right) \right]}{max\left( H\left( U \right),H\left( V \right) \right)-E\left[ MI\left( U,V \right) \right]}$$

where $E[MI(U,V)]$ is the expected value of mutual information, and $max(H(U),H(V))$ is the maximum value of entropy for the true labels and the predicted clusters. The range of values of AMI is $[-1,1]$, with higher values indicating higher agreement between the clusters and the true labels.

**1.3 Homogeneity**

Homogeneity is one of the metrics used to assess the quality of the clustering results, which measures whether each cluster contains only samples from a single category. In other words, homogeneity reflects the “purity” of each cluster in the clustering result. If the samples in each cluster belong to the same category, homogeneity is 1; if the samples in the cluster come from more than one category, homogeneity is lower. Its formula is based on Conditional Entropy:

$$Homogeneity=1-\frac{H\left( C|K \right)}{H\left( C \right)}$$

where $C$ is the true category label, $K$ is the clustering result, $H(C|K)$ is the conditional entropy of the true category label $C$ given the clustering result $K$, and $H(C)$ is the true category label $C$'s entropy.

The conditional entropy $H(C|K)$ is formulated as:

$$H\left( C | K \right)=-\sum_{k=1}^{\left| K \right|} \sum_{c=1}^{\left| C \right|} \frac{n_{c,k}}{n}\log\frac{n_{c,k}}{n_{k}}$$

where $n_{c,k}$ is the number of samples whose true category is $c$ and are assigned to cluster $k$, $n_{k}$ is the total number of samples in cluster $k$, and $n$ is the total number of samples.

The entropy $H(C)$ is calculated by the formula:

$$H\left( C \right)=-\sum_{c=1}^{\left| C \right|} \frac{n_{c}}{n}\log\frac{n_{c}}{n}$$

where $n_{c}$ is the true category of $c$ the number of sample.

**1.4 Moran’s I score**

Moran’s I is a statistical measure of spatial autocorrelation. It is used to assess whether the distribution of a variable in spatial data is aggregated or dispersed. In our experiments, given a feature (gene or label) and the spatial location of the observations, we can use Moran's 𝐼 score to assess whether the expressed pattern is clustered, dispersed, or randomized. We used the Squidpy[1] package to calculate the Moran’s I score.

Moran's I is calculated by the formula:

$$I=\frac{N}{W}\cdot\frac{\sum_{i=1}^{N} \sum_{j=1}^{N} w_{ij}\left( x_{i}-\bar{x} \right)\left( x_{j}-\bar{x} \right)}{\sum_{i=1}^{N} \left( x_{i}-\bar{x} \right)^{2}}$$

where $N$ is the number of samples, $x_{i}$ and $x_{j}$ are the observations of samples $i$ and $j$, $\bar{x}$ is the mean of all sample observations, $w_{ij}$ is the spatial weight between samples $i$ and $j$, and $W$ is the sum of all $w_{ij}$.

1. **Competitive Methods**

We compared SpaMI with three state-of-the-art multi-omics data integration methods, including one spatial multi-omics data integration method SpatialGlue[2] and two single-cell multi-omics data integration methods, Seurat[3] and MOFA+[4]. The details of the methods and implementations were described as follows.

**2.1 SpatialGlue**

SpatialGlue is a deep learning model based on graph neural networks specialized in handling spatial multi-omics data integration tasks, and we followed the tutorials it provides to process all the datasets (<https://spatialglue-tutorials.readthedocs.io/en/latest/>). For SpatialGlue’s data pre-processing of the RNA modality, 2000 and 3,000 highly variable genes were selected for log normalization for the MISAR dataset and the other datasets, respectively. For the RNA & ATAC dataset, the dimensions of feature reduction were set to 50 for both the RNA and ATAC modalities. For the mouse thymus dataset (RNA&ADT), the dimensions of feature reduction were set to 21 for both the RNA and ADT modalities. We used SpatialGlue's recommended hyperparameters for the different datasets.

**2.2 Seurat WNN**

Seurat WNN analysis is an unsupervised framework to learn the relative utility of each data type in each cell, enabling an integrative analysis of multiple modalities. We followed the vignette it provides to process all the datasets (<https://satijalab.org/seurat/articles/weighted_nearest_neighbor_analysis>). For Seurat’s data pre-processing of the RNA modality, 2,000 and 3,000 highly variable genes were selected for log normalization for the MISAR dataset and the other datasets, respectively. For the RNA & ATAC data, the dimensions of feature reduction were set to 50 for both the RNA and ATAC modalities. For the mouse thymus dataset (RNA&ADT), the dimensions of feature reduction were set to 20 for both the RNA and ADT modalities.

**2.3 MOFA+**

MOFA+ (Multi-Omics Factor Analysis+) is a statistical model and tool for multi-omics data integration. It is capable of analyzing multiple histology datasets simultaneously and extracting shared and dataset-specific latent factors from them. These latent factors can explain variation in the data and reveal associations between different histology data. We followed the vignettes it provides to process all the datasets (<https://biofam.github.io/MOFA2/tutorials.html>). For MOFA+, the top 2,000 and 5,000 highly variable genes and peaks were chosen for normalization for the RNA and ATAC modality data, respectively. The rest of the parameters are default values for the MOFA+.

1. **Simulation datasets**

We simulated spatial multi-homology data based on the non-negative spatial factorization method proposed by Townes et al[5]. We chose the 'ggblocks' model among the two ways of simulating data proposed by them to generate expression matrices for different modalities. We generated gene expression data conforming to a zero-inflated negative binomial distribution (ZINB), protein expression data conforming to a negative binomial distribution (NB), and chromatin accessibility data conforming to a zero-inflated Poisson distribution (ZIP). We simulated two scenarios to test the applicability of the model, one with a combination of spatial transcriptome and spatial proteome and the other with a combination of spatial transcriptome and spatial epigenome. Four different factors were included in all datasets as well as backgrounds that could represent five cell types. We generated 4 datasets for each scenario and added varying degrees of Gaussian distributed noise to the datasets to better simulate real-world scenarios and to test the model's noise resistance. The summary statistics for the 8 datasets are shown in Table S1.

**Reference**

1. Palla G, Spitzer H, Klein M et al. Squidpy: a scalable framework for spatial omics analysis, Nat. Methods 2022;19:171-178.

2. Long YH, Ang KS, Sethi R et al. Deciphering spatial domains from spatial multi-omics with SpatialGlue, Nat. Methods 2024;21:1658-1667.

3. Hao Y, Hao S, Andersen-Nissen E et al. Integrated analysis of multimodal single-cell data, Cell 2021;184:3573-3587.e29.

4. Argelaguet R, Arnol D, Bredikhin D et al. MOFA plus : a statistical framework for comprehensive integration of multi-modal single-cell data, Genome Biol. 2020;21:111.

5. Townes FW, Engelhardt BE. Nonnegative spatial factorization applied to spatial genomics, Nat. Methods 2023;20:229-238.

**Table A. Summary information on the simulation dataset.**“mean” and “std” are mean and standard deviation of the Gaussian distribution, respectively.

| Dataset | RNA modality | | | | ADT/ATAC modality | | | |
| --- | --- | --- | --- | --- | --- | --- | --- | --- |
|  | spot | dimension | Gaussion | | spot | dimension | Gaussion | |
|  |  |  | mean | std |  |  | mean | std |
| Simulation1 | 1296 | 1000 | 2 | 0.4 | 1296 | 100 | 2 | 0.4 |
| Simulation2 | 1296 | 1000 | 2 | 0.6 | 1296 | 100 | 2 | 0.6 |
| Simulation3 | 1296 | 1000 | 2 | 0.8 | 1296 | 100 | 2 | 0.8 |
| Simulation4 | 1296 | 1000 | 2 | 1.0 | 1296 | 100 | 2 | 1.0 |
| Simulation1 | 1296 | 1000 | 2 | 0.4 | 1296 | 5000 | 2 | 0.4 |
| Simulation2 | 1296 | 1000 | 2 | 0.6 | 1296 | 5000 | 2 | 0.6 |
| Simulation3 | 1296 | 1000 | 2 | 0.8 | 1296 | 5000 | 2 | 0.8 |
| Simulation4 | 1296 | 1000 | 2 | 1.0 | 1296 | 5000 | 2 | 1.0 |

**Table B. Evaluation of the computational resources required for the datasets**

| Dataset | scales | training time (s/10 epochs) | memory (MB) |
| --- | --- | --- | --- |
| Spatial ATAC–RNA-seq mouse brain dataset | 9215x22914 9215x121068 | 2.35 | about 2300 |
| MISAR-seq mouse embryonic brain dataset | 1949x2144 1949x47287 | 0.93 | about 600 |
| Stereo-CITE-seq mouse thymus dataset | 4697x23622 4697x51 | 1.34 | about 1000 |
| Human Lymph Node Samples dataset | 3484x18085 3484x31 | 1.12 | about 800 |

**Supplemental Figures**


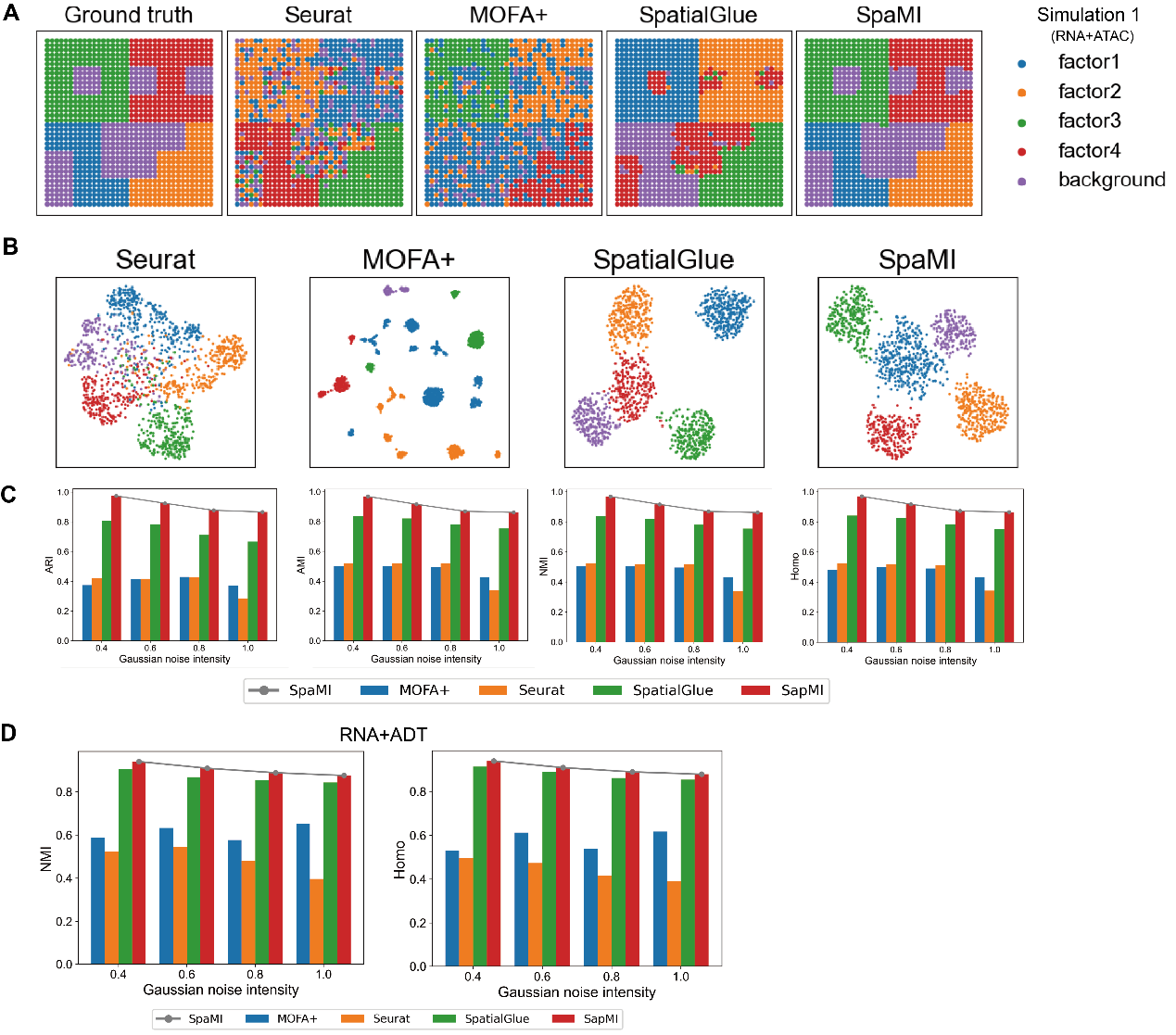


**Figure A. Comparison of SpaMI with other methods on simulated spatial multi-omics datasets (RNA+ATAC).** **(A)** Spatial visualization of Ground truth, Seurat, MOFA+, SpatialGlue and SpaMI on the simulated data (RNA+ATAC). **(B)** UMAP visualization of Seurat, MOFA+, SpatialGlue and SpaMI on simulated data (RNA+ATAC). **(C)** Quantitative evaluation of the four methods using ARI, AMI, NMI and Homogeneity metrics on simulated data (RNA+ATAC). The horizontal axis represents the gradually increasing standard deviation of Gaussian noise. **(D)** Quantitative evaluation of the four methods using NMI and Homogeneity metrics on simulated data (RNA+ADT).


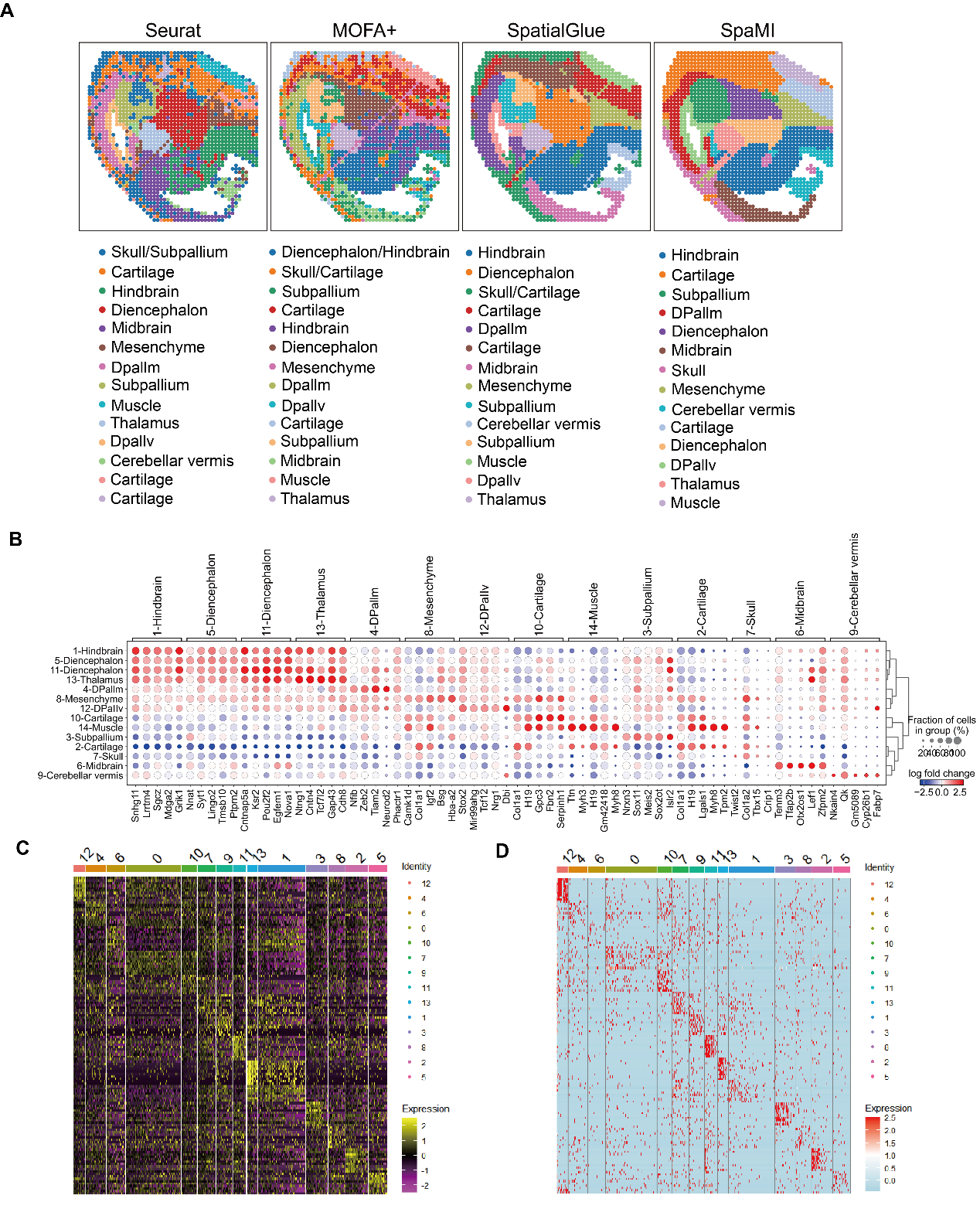


**Figure B. Result for mouse embryonic brains dataset.** **(A)** Spatial visualization of Seurat, MOFA+, SpatialGlue and SpaMI on the mouse embryonic brains data. **(B)** Dot plot for differentially expressed genes on mouse embryonic brains data. **(C)** Heatmap of differentially expressed genes for each cluster for the mouse embryonic brains data. **(D)** Heatmap of differentially expressed peaks for each cluster for the mouse embryonic brains data.


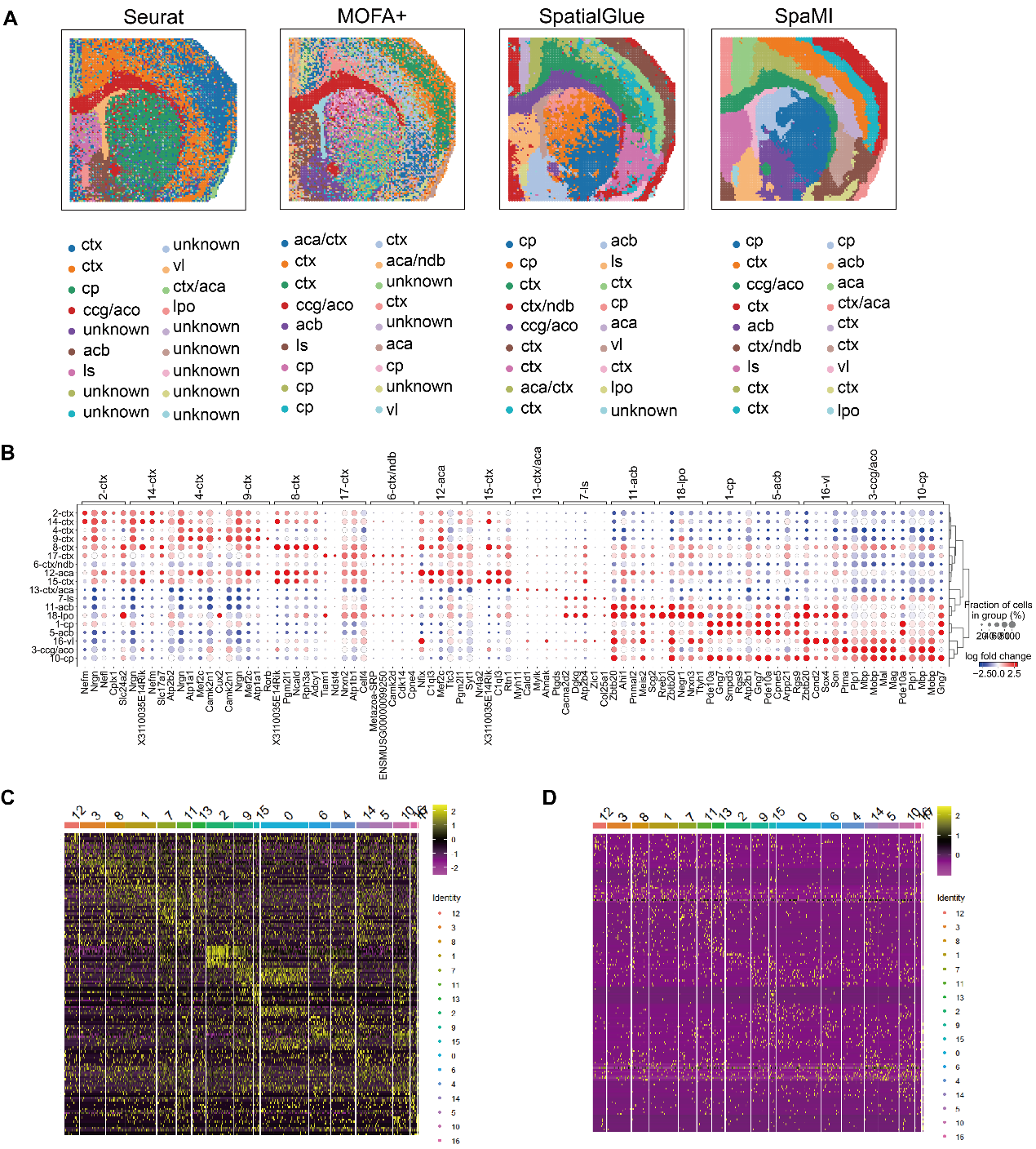


**Figure C. Result for the juvenile mouse brain data. (A)** Spatial visualization of Seurat, MOFA+, SpatialGlue and SpaMI on the juvenile mouse brain data. **(B)** Dot plot for differential genes on juvenile mouse brain data. **(C)** Heatmap of differentially expressed genes for each cluster for the juvenile mouse brain data. **(D)** Heatmap of differentially expressed peaks for each cluster for the juvenile mouse brain data.


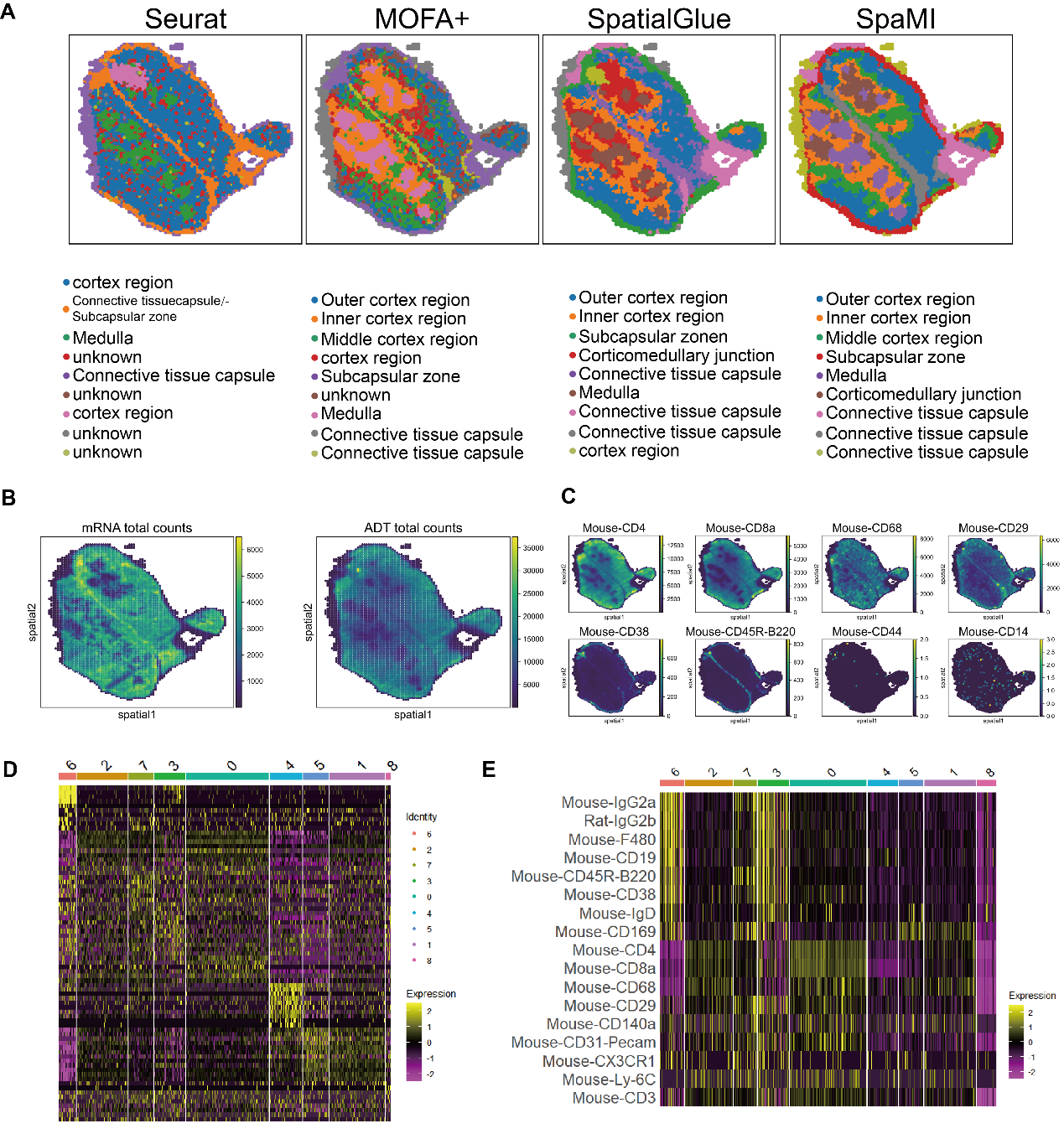


**Figure D. Result for the mouse thymus data.** **(A)** Spatial visualization of Seurat, MOFA+, SpatialGlue and SpaMI on the mouse thymus data. **(B)** Spatial plots of the total mRAN expression and the total ADT counts. **(C)** Intensity plots of some ADTs for the mouse thymus data. **(D)** Heatmap of differentially expressed genes for each cluster for the mouse thymus data. **(E)** Heatmap of differentially expressed ADTs for each cluster for the mouse thymus data.


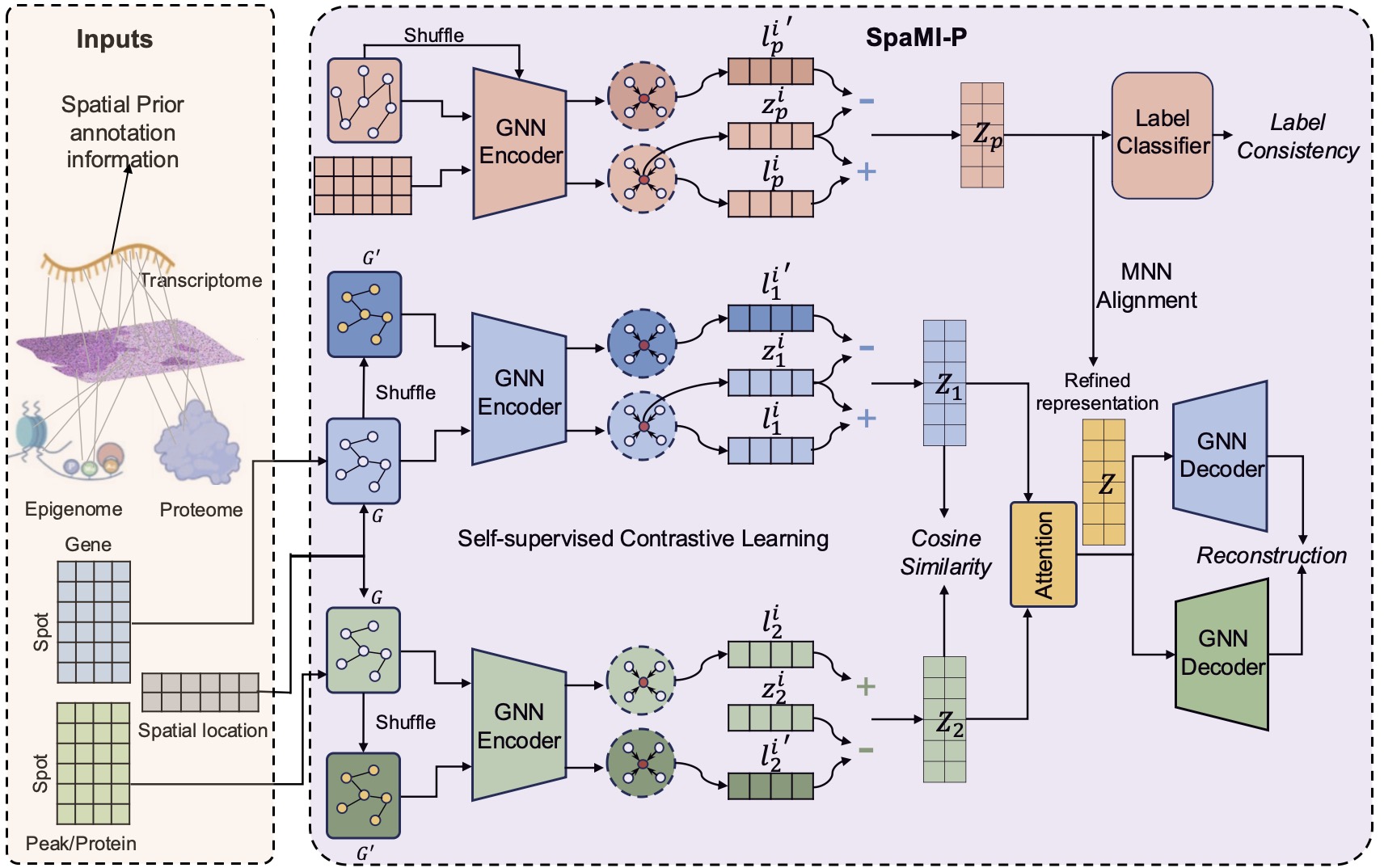


**Figure E. Overview of SpaMI-P.** The inputs of SpaMI-P are spatial multi-omics data including transcriptomic profile, epigenomic profile or proteomic profile, spatial location and spatial genomics with prior annotation information. SpaMI-P first encodes the prior modality data using an omics-specific encoder with the same structure as SpaMI to obtain a low-dimensional embedding, and then align the embedding of the prior data with the embedding of the spatial multi-omics data through the MNN alignment strategy. The embedding of the prior data is then used to predict cell labels through a classifier.


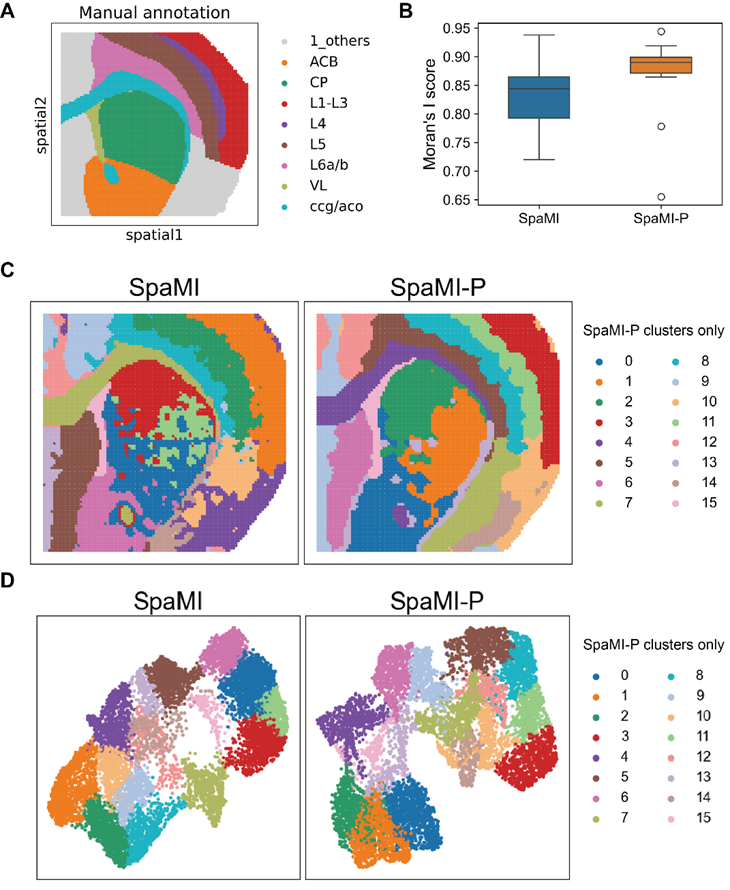


**Figure F. Comparison the results of SpaMI and SpaMI-P on P22 data. (A)** Manual annotation results for the prior data (Spatial ATAC-RNA-seq mouse brain dataset). **(B)** Box plots of Moran’s I score of SpaMI and SpaMI-P. **(C)** Spatial clustering results of SpaMI and SpaMI-P. SpaMI uses RNA+H3K27ac data (CUT&Tag-RNA-seq mouse brain dataset), while SpaMI-P uses RNA+H3K27ac data and incorporates prior data (Spatial ATAC-RNA-seq mouse brain dataset) of ATAC modality. **(D)** UMAP visualization of the results of SpaMI and SpaMI-P.


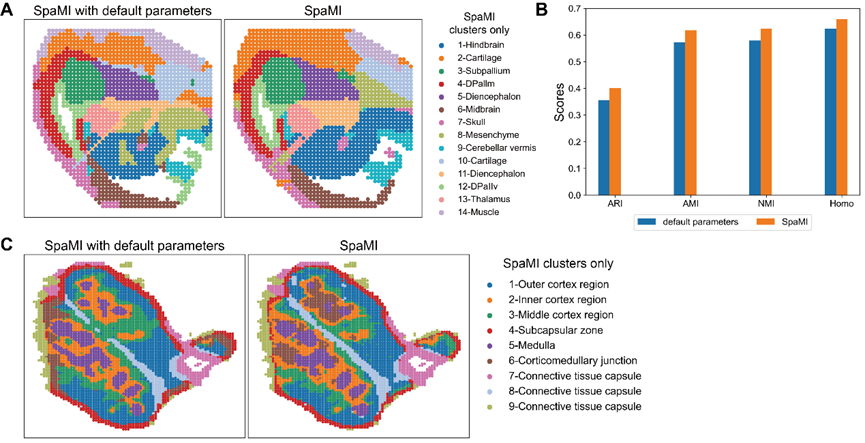


**Figure G.** **(A)** Spatial clustering results of SpaMI on MISAR-seq mouse embryonic brain dataset using default and adjusted parameters. **(B)** Results of quantitative evaluation of SpaMI on MISAR-seq mouse embryonic brain dataset using default and adjusted parameters. **(C)** Spatial clustering results of SpaMI on Stereo-CITE-seq mouse thymus dataset using default and adjusted parameters.


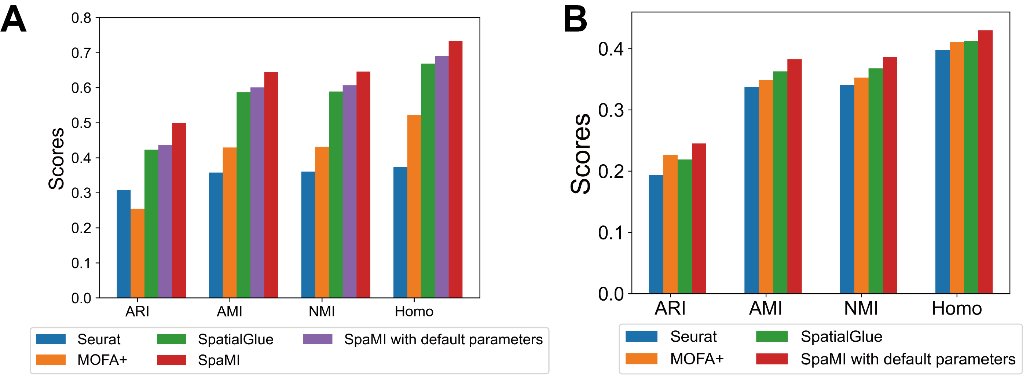


**Figure H.** **(A)** Results of quantitative evaluation on Spatial ATAC-RNA-seq mouse brain dataset. Methods used include Seurat, MOFA+, SpatialGlue, SpaMI and SpaMI with default parameters. **(B)** Results of quantitative evaluation on Human Lymph node dataset.


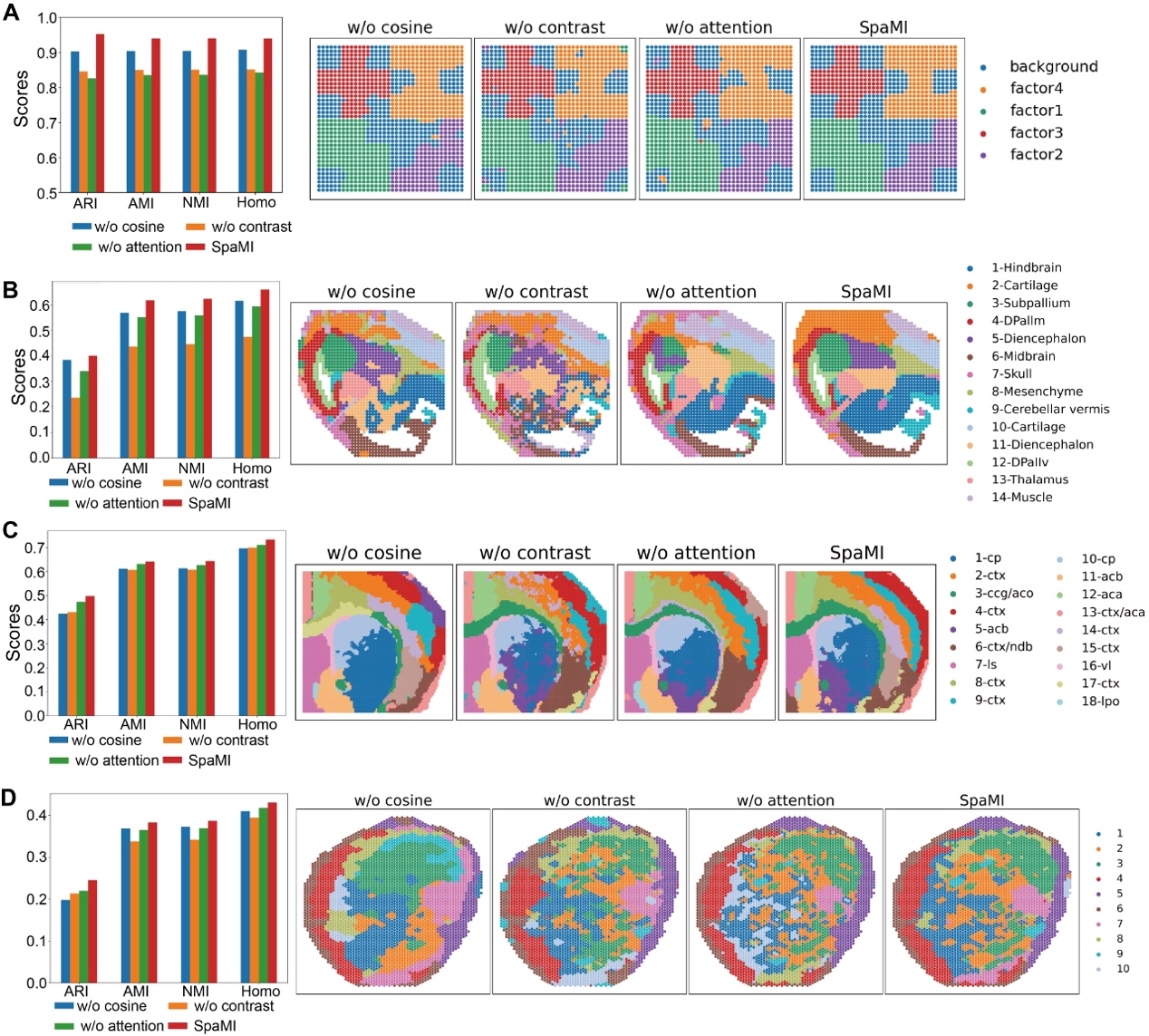


**Figure I.** **Ablation study on four datasets.** **(A)** Barplot of ARI, AMI, NMI and Homo of each ablation experiment on the simulated dataset (left). Spatial Visualization of clusters identified on the embeddings of each ablation experiment on the simulated dataset (right). **(B)** Barplot of ARI, AMI, NMI and Homo of each ablation experiment on MISAR-seq mouse embryonic brain dataset (left). Spatial Visualization of clusters identified on the embeddings of each ablation experiment on MISAR-seq mouse embryonic brain dataset (right). **(C)** Barplot of ARI, AMI, NMI and Homo of each ablation experiment on juvenile mouse brain dataset (left). Spatial Visualization of clusters identified on the embeddings of each ablation experiment on juvenile mouse brain dataset (right). **(D)** Barplot of ARI, AMI, NMI and Homo of each ablation experiment on human lymph node dataset (left). Spatial Visualization of clusters identified on the embeddings of each ablation experiment on human lymph node dataset (right).
