# Supplementary material for: A graph neural network-based spatial multi-omics data integration method for deciphering spatial domains
Source: PLoS Comput Biol. 2025 Sep 30;21(9):e1013546. doi: 10.1371/journal.pcbi.1013546 (PMC12503342; doi:10.1371/journal.pcbi.1013546)
Supplement: Attachment — Submitted filename: Response to Reviewers.pdf [file pcbi.1013546.s004.pdf]

**Response to comments for paper PCOMPBIOL-D-25-00362R1 "A graph neural network-based spatial multi-omics data integration method for deciphering spatial domains"**

**Reviewers' comments:**

**Reviewer #3:**

1. While the authors have quantitatively demonstrated the contributions of each components using simulated data, these results are insufficient. First, no visualization results are provided. Quantitative evaluation alone may not objectively reflect model performance. Visual evaluation should be also included to support the findings. Second, the ablation study is limited to simulated data, which is overly simplistic and may not accurately capture the complexity of real-world scenarios. Therefore, the inclusion of real-world datasets in the ablation study is necessary to more rigorously validate the model' s effectiveness.

**Response:** Thank you for your comment. In the revised manuscript, we have added the spatial visualization results and ablation experiments on other real-world datasets to new Supplementary Figure S9.

2. In their response, the authors noted that prior information such as annotations was used in the extended model. However, this detail appears to be missing from the manuscript. If such supervision was indeed employed, the comparisons with baseline methods may be biased, as many of the baselines are unsupervised. This discrepancy should be clarified to ensure a fair evaluation.

**Response:** We apologize for the confusion. In the revised manuscript, we have added the details of the extended model SpaMI-P to the Results section and Supplementary Figure S4. SpaMI-P was developed to demonstrate that the SpaMI framework is flexible and can be extended to incorporate prior knowledge from public databases. For all comparisons with baseline methods across the datasets, we used the standalone, unsupervised SpaMI model, which does not utilize any annotation information.

3. The figures should be improved. For example, the labels are provided only for the proposed methods, such as Fig. 2A, Fig. 3C. The labels for other methods should be also provided.

**Response:** Thanks for your suggestion. In the revised manuscript, we have updated the figures. We have adjusted the color scheme to ensure that each color consistently represents the same annotated label in Fig. 2A. We also have added the labels for other methods to the Supplementary Fig. S2A, S3A, S4A.
