# Supplementary material for: A graph neural network-based spatial multi-omics data integration method for deciphering spatial domains
Source: PLoS Comput Biol. 2025 Sep 30;21(9):e1013546. doi: 10.1371/journal.pcbi.1013546 (PMC12503342; doi:10.1371/journal.pcbi.1013546)
Supplement: Attachment — Submitted filename: Response to Reviewers_revised.docx [file pcbi.1013546.s003.docx]

**Response to comments for paper PCOMPBIOL-D-25-00362 “A graph neural network-based spatial multi-omics data integration method for deciphering spatial domains”**

**Reviewers' comments:**

**Reviewer #1:**

1. It would be beneficial to briefly discuss the specific biological problems your study aims to address and their potential biological significance in the introduction. This would enhance the relevance and impact of your research.

**Response:** We thank the reviewer for this constructive suggestion. We fully agree that elaborating on the specific biological problems and their significance in the introduction will greatly improve the clarity of our research motivation. In the revised manuscript, we have added a new paragraph in the introduction section to discuss the biological problems our study intends to solve.

2. The literature review section should be further enriched with a more detailed discussion of existing methods, particularly those directly related to the methods proposed in this paper.

**Response:** We sincerely appreciate your insightful feedback. In the revised literature review, we have incorporated additional content to enable a more in-depth and detailed discussion of existing methods.

3. As a computational model, the article does not provide detailed information on the computational resource requirements of the SpaMI model, including runtime and memory consumption.

**Response:** We apologize for missing the detailed computational resource information. In the revised manuscript, we have added the computational resources requirements of the model and the runtime and memory consumption to the “Implementation details and parameter selection” of the manuscript.

4. For datasets without annotations, the authors used only Moran's I as the evaluation metric. Additional metrics should be employed to more comprehensively validate the performance of SpaMI.

**Response:** Thanks for the valuable suggestion. In the revised manuscript, we

have added ARI, AMI, NMI and Homogeneity metrics compared to manually annotated structures. The specific results are demonstrated in updated Figure 3 and updated Figure 4. Compared with other competing methods, our model achieved the highest scores in all metrics.

5. The manuscript contains some textual errors, such as "import" on page 9. Please review the entire manuscript carefully.

**Response:** Thanks for pointing out this point. We have conducted a thorough and meticulous review of the entire manuscript. All spelling mistakes, including the incorrect use of “import” on page 9, have been corrected.

6. Concerns remain about the experimental design. While the authors reported external clustering metrics (e.g., ARI, NMI) for simulated datasets, they only used internal metrics for real datasets. Given the significant differences between simulated and real data, the validation should be strengthened by including more annotated real datasets.

**Response:** Thanks for the insightful suggestion. In the revised manuscript, we have added a new dataset with authentic annotations, which is a human lymph node dataset including spatial transcriptomic and spatial proteomic data. We conducted spatial multi-omics data integration experiments on this dataset using SpaMI and other methods. SpaMI is able to identify the major spatial domains with higher Moran'I value than other methods. Moreover, we also calculated the external clustering metrics such as ARI and AMI for each method using the labels of this dataset. SpaMI had higher values than other competing methods in all four metrics (new Figure 7), further reflecting the advantages of SpaMI in the field of spatial multi-omics data integration.

In addition, we also used the manual annotation labels of the MISAR-seq mouse embryonic brain dataset and the annotation information of the Spatial ATAC-RNA-seq mouse brain dataset given in the previous study (https://doi.org/10.1038/s41467-024-55204-y) as ground truth to calculate ARI and other metrics, and SpaMI also performed well (updated Figures 3 and 4.).

**Reviewer #2:**

1. Benchmarking on Public Datasets:
The authors mention multiple spatial multi-omics technologies (Refs. 7–16). Given the growing availability of public datasets, could SpaMI be tested on additional datasets (e.g., tumor tissues or human samples) to further validate its generalizability?

**Response:** Thanks for the insightful suggestion. In the revised manuscript, we have applied SpaMI to a new human lymph node dataset, which was generated by 10x Genomics Visium RNA and Protein co-profiling technology. In addition, this dataset was manually annotated by Long et al.(https://doi.org/10.1038/s41592-024-02316-4) so that we can quantitatively evaluate the performance of each method by calculating supervised metrics such as ARI and AMI. The experimental results on this dataset are shown in new Figure 7. SpaMI is able to identify the major spatial domains with higher Moran'I value than other methods. We also calculated the external clustering metrics such as ARI and AMI for each method using the labels of this dataset. SpaMI had higher values than other competing methods in all four metrics, further reflecting the advantages of SpaMI in the field of spatial multi-omics data integration.

2. Estimation of Spatial Domain Number:

How is the number of spatial domains determined? Could automated methods (e.g., ASTER, scCCESS) be integrated to estimate domain numbers, similar to cell-type quantification in single-cell analysis?

**Response:** Thanks for the comments. In our experiments, the number of spatial domains was similar to the number of annotated spatial structures. For example, when determining the number of spatial domains in the Spatial ATAC-RNA-seq mouse brain dataset, we referred to the spatial structure of the corresponding biological tissue provided by the Allen brain atlases. In addition, we applied ASTER to the Spatial ATAC-RNA-seq mouse brain dataset. Specially, we treated the reconstructed spatial epigenomic data obtained by the SpaMI model as input of the ASTER method. The method determined 18 as the output result in the range from 15 to 20, which was consistent with the number of spatial domains we used in the manuscript. Moreover, we applied ASTER to the MISAR-seq mouse embryonic brain dataset. The estimated number was 10 from 8 to 15, while the number of spatial domains we used in the manuscript was 14. This might because that spatial genomics data had large noise level than single cell data. Such methods can still provide a prior information on the number of spatial domains.

3. Prior-Based Learning:

Public databases (e.g., 10x Visium, Allen Brain Atlas) host vast prior data. Can SpaMI leverage such resources to enhance integration via transfer learning or guided training (10.1101/gr.277891.123)?

**Response:** Thank you for the insightful suggestions. SpaMI can leverage such prior data. In the revised manuscript, we have extended SpaMI to SpaMI-P, which is an extension of SpaMI that incorporates a prior data incorporation module into its framework.

Specifically, SpaMI-P first encodes the prior modality data using an omics-specific encoder with the same structure as SpaMI to obtain a low-dimensional embedding, and then align the embedding of the prior data with the embedding of the spatial multi-omics data through the MNN alignment strategy. The embedding of the prior data is then used to predict cell labels through a classifier. Then we trained the priori data incorporation module with the original spatial multi-omics data integration model together. Next, we applied SpaMI-P to the Spatial ATAC-RNA-seq mouse brain dataset, which contained RNA-seq and CUT&Tag (acetylated histone H3 Lys27 (H3K27ac) histone modification) modalities. And we used the ATAC modality data in the Spatial ATAC-RNA-seq mouse brain dataset as prior data, which had manually annotated labels. We found that SpaMI-P well divided the cortical layer than SpaMI. And SpaMI-P had higher Moran’s I score than that of SpaMI. These experimental results well illustrate that prior information play an important role in guiding the model training process. In the revised manuscript, we have added these results to Result section and the figures S4-S5.

4. Scalability via Metacell Strategies:

With increasing data scale, could metacell-based approaches (e.g., EpiCarousel) be adopted to improve computational efficiency while preserving spatial resolution?

**Response:** Thanks for the valuable suggestion. I agree with the reviewer that metacell-based approaches could improve computational efficiency. However, it cannot preserve the spatial resolution. It might be not suitable for the spatial genomics data, which do not have spatially continuous patterns such as the spatial data of tumor with the existence of infiltrated immune cells.

5. Computational Efficiency:

The manuscript critiques spaMultiVAE’s complexity. Could the authors quantify SpaMI’s runtime, memory usage, and scalability on larger datasets?

**Response:** We apologize for missing the detailed computational resource information. In the revised manuscript, we have added the computational resources requirements of the model and the runtime and memory consumption to the “Implementation details and parameter selection” of the manuscript.

6. Tool Packaging for Accessibility:

While the GitHub repository is valuable, packaging SpaMI into a Python library (e.g., via PyPi) with standardized APIs and tutorials would greatly enhance its usability and adoption.

**Response:** Thanks for the suggestion. In the revised manuscript, we have packaged SpaMI as a Python library and released it on PyPi. Users can install it through the "pip install SpaMI" command, and we have also updated the tutorials.

**Reviewer #3:**

1. Insufficient novelty. SpaMI combines graph neural networks (GNN) with attention mechanism, which is quite similar to the approach in Long et al., 2024. Although contrastive learning is incorporated in SpaMI, the difference between the two methods is minimal

**Response:** We thank the reviewer for the comments. Although both SpatialGlue and spaMI adopted graph neural networks (GNN) and attention mechanisms, there are major differences between spaMI and SpatialGLUE. There are three parts in SpaMI model, which are contrastive learning strategy, attention mechanism and cosine similarity regularization. We have conducted ablation studies to validate that each part improves the integration performance. The contrastive learning strategy was added to the graph neural network to extract spatially informative features. In addition, we added cosine similarity regularization to better extract the homogeneity between different omics. In the revised manuscript, we have extended spaMI model, which can incorporate the prior information such as annotation to guide the training process. Overall, SpaMI is different to the approach in Long et al. 2024. In the revised manuscript, we have clarified this point in the Discussion section.

2. Weak results. The authors evaluated the performance of the proposed method using simulated and three real spatial multi-omics data. However, SpaMI appears comparable to the baseline, SpatialGlue, across most datasets. For example,

 Visually, the spatial distribution and UMAP results for the simulation show that SpaMI and SpatialGlue are comparable. Both methods accurately identify the four factors and background.

 In the mouse thymus results, it is difficult to discern any significant advantage of SpaMI over SpatialGlue, as both methods identify main tissue regions with high accuracy.

**Response:** Thanks for the comments. In the revised manuscript, we have added the detailed quantitative comparisons with annotation labels. SpaMI had higher values than SpatialGLUE in all the metrics across most datasets.

I agree with the reviewer that on the first simulated dataset, both SpaMI and SpatialGlue could accurately identify the four factors and background. On the second simulated RNA+ATAC data, SpatialGLUE failed to detect the factor edges, while spaMI accurately detected the factors and background (Supplementary Figure S1).

In the mouse thymus dataset, both SpaMI and SpatialGlue methods could identify major tissue regions, such as the medulla and cortex. However, SpaMI was able to capture the spatial structure of tissues in a more fine-grained manner. Specially, SpaMI could identify three cortical layers of structure: inner, middle, and outer, while SpatialGlue only identifies two layers of cortical structure. In the revised manuscript, we have clarified them in the Result section.

3. Inaccurate or unclear descriptions:

 The author claim that single cell multi-omics integration methods might not be applicable for spatial multi-omics data as spatial information is not considered. However, these methods can still be applied to spatial multi-omics data, as one can choose not to use spatial information.

 The author state, “However, the model adopts single-layer GCN as encoder, making the network structure too simple to effectively capture complex structures”. Are there any supporting evidence or results for this claim?

**Response:** Thank you for the comments. In the revised manuscript, we have updated the unclear descriptions.

4. Missing labels. Some figures lack labels, such as Fig.3 C, E, Fig. 4 C, E, Fig. 6 C, F. The authors provide labels for SpaMI, but not for other methods.

**Response:** Thanks for the comments. In the revised manuscript, we have updated the figure caption and added the figures with labels to the supplementary figures.

5. How about the robustness of the proposed method? Most parameters vary across different datasets, which might weaken the generalizability of the proposed method when applied to new datasets.

**Response:** Thanks for the comments. In the revised manuscript, we provided a default parameter set for the model, which could be applied to most of the datasets. In addition, we compared the performance of SpaMI using default parameters with other methods on spatial ATAC-RNA-seq mouse brain data, which had reliable annotation based on the Allen brain atlases. We found that SpaMI still had better metric values than other methods (new Supplementary Figure S7).

6. Some typos can be found in the manuscript, such as “is decrease”.

**Response:** Thank you for pointing it out. We sincerely apologize for the typos in the manuscript. We have checked the entire manuscript thoroughly and carefully. All spelling errors, including the incorrect use of the word “is decrease” on page 7 of the manuscript, have been corrected.
